# Reductions in perceived COVID‐19 threat amid UK’s mass public vaccination programme coincide with reductions in outgroup avoidance (but not prejudice)

**DOI:** 10.1111/bjso.12537

**Published:** 2022-03-31

**Authors:** Rose Meleady, Gordon Hodson

**Affiliations:** ^1^ School of Psychology University of East Anglia Norwich UK; ^2^ Department of Psychology Brock University St. Catharines Ontario Canada; ^3^ Forensic Psychology and Criminal Justice (FPAC) Programme Brock University St. Catharines Ontario Canada

**Keywords:** behavioural immune system, COVID‐19, pathogen avoidance, prejudice, threat, vaccination

## Abstract

It has long been proposed that perceptions of threat contribute to greater outgroup negativity. Much of the existing evidence on the threat–prejudice association in the real world, however, is cross‐sectional in nature. Such designs do not adequately capture individual‐level *changes* in constructs, and how changes in constructs relate to changes in other theoretically relevant constructs. The current research exploited the unique opportunity afforded by the mass COVID‐19 vaccination programme in the United Kingdom to explore whether reductions in pathogen threat coincide with reductions in outgroup prejudice and avoidance. A two‐wave longitudinal study (*N*_1_ = 912, *N*_2_ = 738) measured British adult's perceptions of COVID‐19 threat and anti‐immigrant bias before and during mass vaccine rollout in the United Kingdom. Tests of latent change models demonstrated that perceived COVID‐19 threat significantly declined as the vaccine programme progressed, as did measures of outgroup avoidance tendencies, but not prejudiced attitudes. Critically, change in threat was systematically correlated with change in outgroup avoidance: those with greater reductions in perceived COVID‐19 threat were, on average, those with greater reductions in outgroup avoidance. Findings provide important and novel insights into the implications of disease protection strategies for intergroup relations during an actual pandemic context, as it unfolds over time.

## INTRODUCTION

A diverse array of social psychological research has established that threatening events, either at a personal or collective level, can produce attitudinal and behavioural shifts in domains not directly related to the immediate situation, including exacerbating prejudice towards outgroups such as immigrants and ethnic minorities (Jonas et al., 2014; Stephan & Stephan, 2000; Xu & McGregor, 2018). Presently, COVID‐19 represents a major threat to human health. The current research exploited the pandemic context to explore evidence for a threat–prejudice association. A unique longitudinal dataset explored the degree to which changes in perceived COVID‐19 threat and anti‐immigrant bias (prejudice; behavioural avoidance tendencies) co‐occurred amid a period of mass public vaccination in the United Kingdom. If outgroup aversion constitutes a defensive reaction to threat, it was expected that reductions in perceived COVID‐19 threat would coincide with reductions in negative attitudes and avoidant behavioural tendencies towards immigrants. Such a design, capturing real‐life pandemic threat reactions, would be a unique contribution to the literature, able to assess whether *changes* in perceived threat coincide with *changes* in outgroup biases (e.g., prejudice or avoidance), and the magnitudes of such relations. Addressing this question could provide important insights into whether public health interventions that target disease‐related concerns may also have beneficial consequences in areas that extend beyond immediate health‐related domains, such as intergroup relations.

### The role of threat in intergroup relations

Realistic group conflict theory (RGCT, Sherif & Sherif, 1969, see also Levine & Campbell, 1972) represents one of the earliest approaches to consider threat in relation to tense or competitive intergroup relations. According to realistic group conflict theory, when two groups feel that they are in competition for finite resources, such as money, political power, or social status, the potential success of one group threatens the well‐being of the other leading to prejudice towards the competing outgroup. Supportive evidence has shown that perceptions of incompatible goals and competition leads to intergroup antagonism, stereotypes, and conflict (e.g., Bettencourt et al., 1992; Sherif et al., 1961; Sidanius et al., 2007; Zarate et al., 2004). More recently, intergroup threat theory (ITT, Stephan et al., 2016, previously known as integrated threat theory, Stephan & Stephan, 2000) has distinguished between realistic and symbolic threats. Realistic threats are threats to the ingroups’ physical, political, or economic well‐being, what are often referred to as ‘tangible’ threats. Symbolic threat, meanwhile, concerns threats to the ingroup's identity, norms, and values. Both types of threat can be experienced at either the group level where the threat is to the ingroup as a whole, or at the individual level where individuals experience threat by virtue of their group membership (Stephan & Renfro, 2002). Furthermore, ITT emphasizes the mere *perception* that an outgroup threatens the self or the ingroup can have negative intergroup consequences (Stephan et al., 2016). Numerous studies based on ITT and its reformulations have shown that both realistic and symbolic threat can trigger negative emotions, perceptions, and behavioural responses towards outgroup members across a range of intergroup contexts (for reviews see Riek et al., 2006; Stephan et al., 2016).

Cottrell and Neuberg’s (2005) sociofunctional model of prejudice is a more recent theory of group threat. This model elaborates on the consequences of threat, suggesting that specific intergroup threats elicit different patterns of adaptive (or ‘functional’) responses. The theory describes a series of different threat‐emotion‐behaviour profiles. The ‘obstacle‐anger‐aggression’ profile, for instance, suggests that when an outgroup is perceived to pose a threat to the ingroup's economic resources or property, anger will and this emotional reaction instigates confrontation behavioural designed to remove the obstacle to resources. The ‘safety‐fear‐escape’ profile suggests that outgroups that poses a threat to the ingroups’ physical safety will elicit fear, prompting escape behaviours. Meanwhile, the ‘contamination‐disgust‐rejection’ profile describes how an outgroup perceived as a source of physical or moral contaminant will evoke disgust, leading to rejection or avoidant behaviours. Research has shown that different threat‐emotion profiles are indeed related to different behavioural intentions towards outgroups (e.g. Johnson & Glasford, 2014; Kamans et al., 2011) as well as different policy attitudes (Cottrell et al., 2010).

Importantly, the models reviewed above focus on *intergroup threats* whereby the source of threat is another group's actions, beliefs, or characteristics, and the threat response (i.e., prejudice) are directed at that group (see Riek et al., 2006). Of interest to the current research is how threats that are seemingly unrelated to intergroup relations can also have implications for intergroup outcomes. In social psychology, application of the term ‘threat’ is broad and can refer any stimulus that may undermine physical or psychological well‐being (e.g., ostracism, loss of control, meaningless, goal conflicts, for review see Jonas et al., 2014). Indeed, the main criterion for determining whether a stimulus is threatening has been its ability to elicit seemingly irrational defensive responses (Xu & McGregor, 2018). Mortality salience, for instance, has been shown to increase ingroup favouritism and outgroup derogation. Greenberg et al. (1990) primed half of their Christian participants with thoughts of their own death prior to forming impressions of Christian and Jewish targets. The manipulation led to more positive evaluations of Christian targets and more negative evaluations of the Jewish targets. Experimentally induced personal uncertainty has also been shown to evoke distancing from socially deviant outgroups (e.g., homeless people; van den Bos et al., 2007; see also Hogg et al., 2007), and meaningfulness threats, such as boredom, can lead people to advocate harsher punishments for outgroup offenders (van Tilburg & Igou, 2011). Whilst these different theoretical approaches emphasize different core needs that are undermined by threats, common to all of them is the notion of ‘fluid compensation’ whereby the threat response does not have to occur in same domain as the threat itself. Such responses can be considered distal, or compensatory threat defences, which do not necessarily tackle the threat at hand but rather attempt to palliate threat‐induced anxiety (see Jonas et al., 2014; Xu & McGregor, 2018).

### Pathogen threat and outgroup prejudice

Since its emergence in late 2019, the COVID‐19 pandemic has had a devastating global impact, claiming the lives of millions of people, and forcing millions more into prolonged periods of home confinement in an attempt to minimize the spread of infections. Research on the behavioural‐immune system (BIS) explores psychological responses to pathogen threats (e.g. Murray & Schaller, 2016; Neuberg et al., 2011; Schaller, 2011; Schaller & Park, 2011). The BIS is a suite of adaptive psychological mechanisms that promote pathogen avoidant behaviour. Because physiological immunological defences are metabolically costly and merely reactionary, the BIS presumably evolved to prevent pathogens entering the body in the first place. One adaptative tendency is the avoidance of individuals displaying symptoms of contagious disease. Such disease‐avoidance mechanisms, however, also provide a foundation for broader prejudices. Because they have evolved to be hypersensitive and risk‐adverse, disease‐avoidance mechanisms can trigger negative attitudes and avoidance behaviour towards targets who do not represent genuine sources of disease (Oaten et al., 2011; Park et al., 2007; Schaller et al., 2003; Schaller & Neuberg, 2012, see also Pond et al., 2011 and Molho et al., 2017 for research linking disgust with reduced aggressive approach tendencies). Research has shown that perceived disease vulnerability increases prejudice towards outgroups including foreigners and immigrants both when measured as a chronic individual difference variable (e.g. Hodson & Costello, 2007; Navarrete & Fessler, 2006) and when experimentally primed (e.g., Faulkner et al., 2004).

Recent research has explored the consequences of COVID‐19 threat with regards to intergroup relations (see Schaller et al., 2021). With the virus thought to have originated in China, initial studies demonstrated positive correlations, in cross‐sectional datasets, between perceived COVID‐19 threat and anti‐Asian attitudes and support for discriminatory Chinese restrictions (Alston et al., 2020; Reny & Barreto, 2020). However, COVID‐19 threat has also been shown to lead to negative perceptions of stigmatized outgroups that are not directly associated with the virus. Meleady et al. (2021) for instance, found that higher subjective COVID‐19 infection estimates were associated with greater preferred physical distance from ethnic minorities generally. Similarly, in Turkey, perceived COVID‐19 threat was indirectly associated with higher anti‐immigrant attitudes and lower support for pro‐immigration policies (Adam‐Troian & Bagci, 2021). Meanwhile, in Italy, Fuochi et al. (2021) found that perceived COVID‐19 threat was associated with lower perceptions of a shared common identity with both national outgroups (e.g., German, Spanish, French, English), and disadvantaged outgroups (e.g., homeless people, people with mental health problems, drug addicts). Relatedly, in a cross‐sectional dataset, Hartman et al. (2020) demonstrated that the association between right‐wing authoritarian (RWA) and anti‐immigrant attitudes in the United Kingdom and the Ireland was stronger amongst those scoring higher in perceptions of COVID‐19 threat suggesting that threats may moderate the effects of authoritarian predispositions.

Importantly, if pathogen threats are associated with prejudicial responses, it follows that these responses may be muted or attenuated to the extent that the threat of contagion can be reduced by disease protection strategies. Hartman et al. (2020) tested whether two modern methods of disease protection could attenuate outgroup prejudice: vaccinations and handwashing. To prime disease threat, some participants read some text about seasonal flu; the disease prime resulted in higher prejudice towards immigrants only amongst unvaccinated participants, and not amongst participants who were protected from the disease. In a second study, prior to reading a disease threat prime, participants in the experimental condition were asked to evaluate a cleansing wipe after using it to wash their hands. The wipe was simply evaluated, without using it, in the control condition. Higher chronic germ aversion was associated with unfavourable outgroup attitudes only in the control condition and not amongst those in the experimental (cleansing) condition. Moreover, effects were observed only for ratings of outgroup members and not ingroup members suggesting that disease protection methods affect prejudice towards outgroups rather than towards people in general. The present research is the first, to our knowledge, to explore the implications of disease protection strategies for outgroup prejudice amid an actual pandemic context, as it unfolds over time.

### The present research: exploring changes in threat

There is now accumulating evidence that the perception of threat, including pathogenic threats, are detrimental to intergroup relations. Much of the existing literature relies, however, on cross‐sectional data (see Riek et al., 2006). Cross‐sectional data provides only a snapshot at a given point of time and cannot directly represent change. Many constructs are time‐varying, necessitating an exploration of fluctuations in variables over a period of change of interest. Of particular interest can be whether parallel changes in variables unfold over time, allowing researchers to determine the degree to which theorized change in one variable is systematically associated with variation in another (Allemand & Martin, 2016). This study employed a unique longitudinal dataset to explore evidence of correlated change in perceived COVID‐19 threat and anti‐immigrant attitudes and behaviour in the context of the UK’s mass COVID‐19 vaccination programme. By focusing on immigrants as the target outgroup, we aimed to explore whether there is an association between threat and outgroup negativity that is not specific to the source of threat but rather generalizes towards ‘others’.

The United Kingdom became the first country in the world to approve a COVID‐19 vaccine in early December 2020. What followed was Britain's largest ever mass vaccination programme. Following guidance devised by Joint Committee on Vaccination and Immunisation (JCVI) Phase 1 of the UK vaccine rollout prioritized care home residents, adults aged 70+, health and social care workers, and the clinically extremely vulnerable. The target of offering a vaccination to all 15 million people in these top priority groups was achieved on the 14th February, 2021. Our data collection coincided with the start of Phase 2, which was a phase of mass vaccination designed to reach the rest of the adult population (Time 1). Participants were contacted again in mid‐April, at which point more than half of all UK adults – some 32 million people – had received their first dose (Time 2). Using a latent change score modelling framework (e.g. Coman et al., 2013; Ghisletta & McArdle, 2012; Henk & Castro‐Schilo, 2016; Hounkpatin et al., 2018; Kievit et al., 2018; McArdle, 2009; Petscher et al., 2016), we explored cross‐domain relations between perceived COVID‐19 threat and anti‐immigrant bias. Latent change models address the nature of change and how change processes are interrelated (i.e., change‐to‐change relations; Henk & Castro‐Schilo, 2016). Our model simultaneously explored longitudinal changes in perceived COVID‐19 threat, anti‐immigrant bias (prejudiced attitudes, behavioural avoidance), and their interrelations over time lags. We expected to observe mean‐level changes in perceived threat and anti‐immigrant bias over time as the vaccination programme progressed, such that both threat and outgroup bias would be lower at Time 2 relative to Time 1. Further, it was expected that change in threat would be correlated with change in outgroup bias such that individuals who displayed larger reductions in perceived COVID‐19 threat over time would also show larger reductions in outgroup bias over time. Our aim was not to infer directionality of relationships between variables such that Time 1 levels of one variable predict Time 2 levels of another variable (i.e., a cross‐lagged model). Rather, we were interested modelling mean‐level changes in variables over time, and to explore the degree to which *changes* in COVID‐19 threat and *changes* in prejudice co‐occur (see Usami et al., 2016 for discussion of model selection between autoregressive cross‐lagged models and latent change score models).

## METHODS

### Participants

Time 1 data collection was conducted on the 22nd of February 2021. All participants completed the Time 1 survey on the same day. We recruited 1003 participants via an online participant panel Prolific. Such recruitment platforms tend to be more demographically diverse than participants recruited via undergraduate student panels, and generally produce results comparable to those from nationally representative samples (Coppock, 2019). The Time 1 sample included 369 male and 630 female participants (4 participants reported their sex as ‘other’), aged between 18 and 76 (*M* = 36.79, *SD* = 13.40). A total of 80.35% of the initial sample returned to complete an identical questionnaire at Time 2 between the 23rd April and the 3rd May 2021. Given our focus, ethnic majority group member's prejudice towards minority groups data from 8 non‐White British participants were excluded. Data were also excluded from participants who failed attention screens or indicated that they did not want their data to be used at either time point. The final sample consisted of 912 participants at Time 1 and 738 participants at Time 2. Only 14.6% of participants had a COVID‐19 vaccination at Time 1 (0.3% of participants had had two doses), a figure that rose to 46.20% at Time 2 (only 8.67% had had two doses). Methods and hypotheses were preregistered at https://aspredicted.org/blind.php?x=dq8ar31^1^

### Measures

The order of all measures was randomized across participants.

#### Perceived COVID‐19 threat

Perceived COVID‐19 threat was measured with a single item, ‘In general, how anxious are you about the COVID‐19 pandemic?’ (Hartman et al., 2020). Participants responded on a slider scale from 0 = Not at all anxious’ to 100 = ‘Extremely anxious’.

#### Outgroup distancing

Outgroup avoidance tendencies were measured in two ways. First, participants’ preference for physical distance between themselves and immigrants was assessed with a pictorial measure adapted from Sorokowska et al. (2017). Participants saw two human‐like figures. Person A was said to represent the self, and Person B an immigrant to the United Kingdom. Participants indicated how close they could approach Person B and feel comfortable having a conversation with them by dragging a slider from the self towards the other. Responses ranged from 0 to 100. Higher scores corresponded to greater outgroup avoidance.

#### Outgroup contact comfort

As a second measure of outgroup avoidance participants indicated their comfort engaging in a series of close contact behaviours with immigrants with a scale adapted from Tybur et al. (2020). Behaviours included ‘Sitting next to them on public transport’, ‘Handling items they had touched’ and ‘Shaking their hand’ (1 = *very uncomfortable* to 7 = *very comfortable*). Nine items were combined to create a single composite score (αs =.93 and.92 at T1 and T2, respectively). Lower scores indicated higher outgroup avoidance.

#### Modern racism

To measure prejudiced attitudes, participants completed the Modern Racism Scale (McConahay et al., 1981) adapted to the immigrant target group. Participants completed 7 items, including ‘Discrimination against immigrants is no longer a problem in Britain’, and ‘Immigrants should not push themselves where they are not wanted’ (1 = *strongly disagree* to 5 = *strongly agree*, αs = .91 and .92 at T1 and T2, respectively). Higher scores indicated higher outgroup prejudice.

#### Ingroup attraction

Participants also completed two feeling thermometer items (adapted from Haddock et al., 1993) to indicate their feelings towards (a) other White Brits, and (b) their own friends and family. Participants indicated how cold (unfavourable) or warm (favourable) they felt towards each group, in general, on a scale from 0° to 100°. (Spearman‐Brown coefficient = .62 and .65 at T1 and T2, respectively).

#### Demographics

Finally, participants provided demographic information, including age, sex, vaccination status, and political orientation. Political orientation was assessed with a single self‐placement item from 1 (*very liberal*) to 7 (*very conservative*).

## RESULTS

### Analytic strategy

**FIGURE 1:**
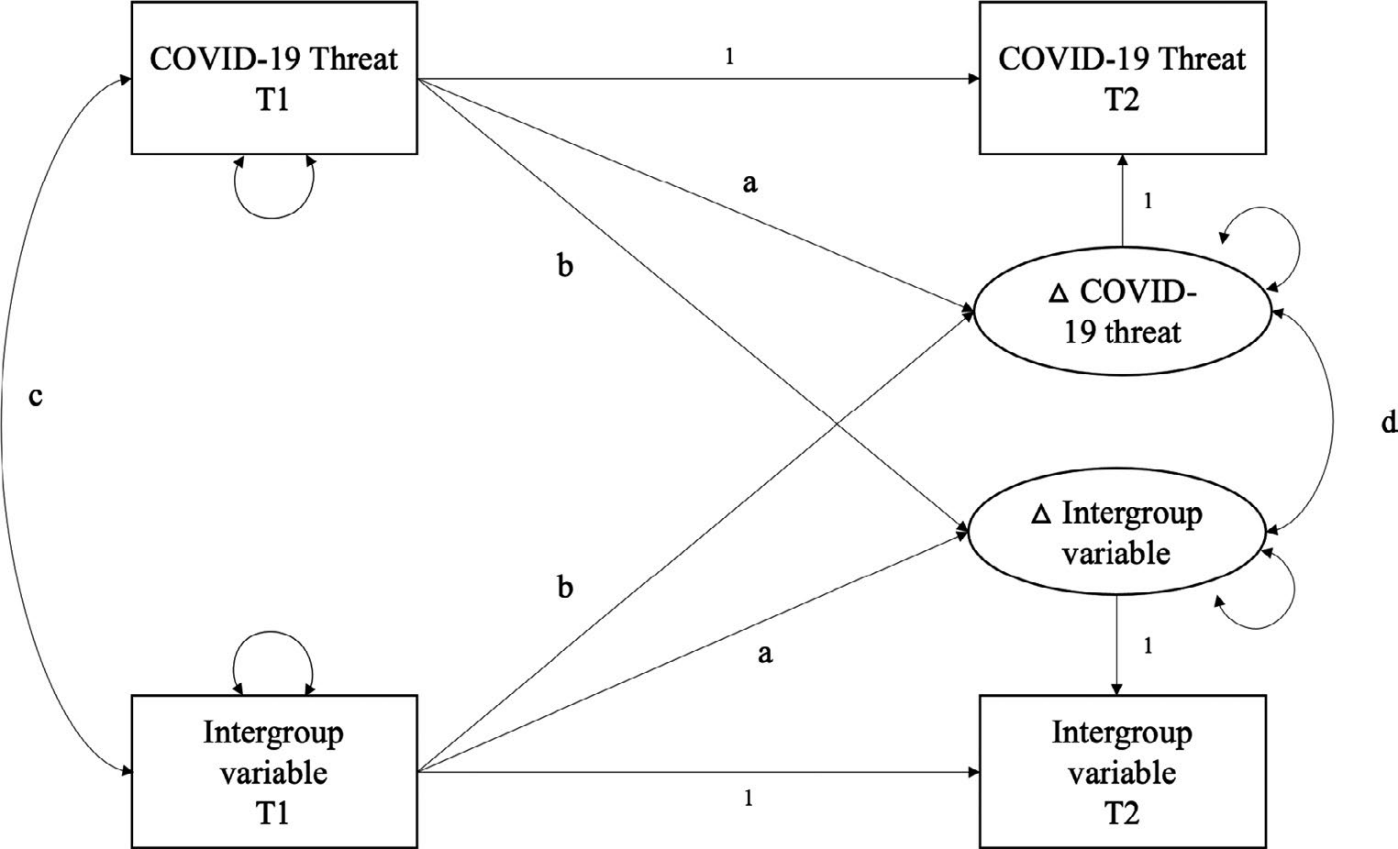
Conceptual depiction of a BLCS model. Parameters a and b = regression of latent change scores on initial values. Parameter c = correlation among baseline values, and Parameter d = correlation among latent change scores

To reduce the undue influence of severe outliers, values greater than 3 SD from the mean were winsorized to the variable's value at 3SD (see Wilcox, 2011). This method of transformation was chosen a priori as part of our pre‐registration. To test hypothesized longitudinal effects, we used latent change score (LCS) models tested within the Lavaan package (Rosseel, 2012) in R software v.4.0.2. Analysis code was adapted from Kievit et al. (2018). LCS models allow researchers to model differences in variables over time using a structural equation modelling framework. Change is modelled as a latent factor which is not directly measured but is explicitly defined as ‘the part of the score of the variable at Time 2 that is not identical to the score of the variable at Time 1’ (McArdle, 2009, p. 583). This approach allows researchers to go beyond traditional methods (e.g., paired samples t‐tests) to estimate a number of additional parameters (McArdle, 2009). The intercept of the latent change factor indicates the rate of change in a variable over time. A significant positive intercept indicates that, on average, scores increased from Time 1 to Time 2, whereas a significant negative intercept indicates that scores decreased over time. The variance/residual variance of the change between Time 1 and Time 2 can also be estimated; this captures the extent to which individuals differ in the change they manifest over time. A significant variance/residual variance of the LCS factor indicates that individuals change heterogeneously, or differently, over time. Mean scores at Time 1 are also modelled along with the covariance between Time 1 scores and the latent change factor indicating the degree to which mean‐level change in a variable is dependent on, or proportional to starting values on that variable (known as a ‘self‐feedback’ pathway, parameter ‘a’ in Figure 1; McArdle, 2009). For instance, a significant negative association would indicate that individuals with higher starting values in variable X at Time 1 show less change in that same variable over time.

Of relevance to the current investigation, a second domain of interest can also be added to LCS model extending the basic univariate LCS model to a bivariate latent change score model (BLCS). BLCS models allow us to explore the extent to which changes in two variables co‐occur (Parameter ‘d’ in Figure 1). That is, the correlation among latent change scores. BLCS models can also estimate the covariance between variable X and variable Y at baseline (Parameter ‘c’ in Figure 1) and the cross‐domain coupling pathways which quantify the extent to which change in one variable is a function of the starting level in the other (Parameter ‘b’ in Figure 1, McArdle, 2009). This is the approach adopted here. A series of BLCS models were estimated for perceived COVID‐19 threat and each of the intergroup variables (outgroup distancing, outgroup contact comfort, modern racism, and ingroup attraction), and the association among change scores tested for significance.^2^ The key parameter of interest to the present project is the correlation between the threat and intergroup variable latent change scores (Parameter ‘d’ in Figure 1).

### Main analyses

Basic descriptives and inter‐correlations amongst all variables at both timepoints are reported in Table 1. Little’s (1988) Missing Completely at Random (MCAR) test was non‐significant, χ^2^(5) = 4.01, *p* = .548, indicating no multivariate differences between those who completed the questionnaire at both time points or only the first. Therefore, the latent change score models were therefore estimated using Full Information Maximum Likelihood (FIML) estimation. Variables were centred prior to being entered into the models. For each variable, the T1 and T2 mean scores were centred on the Time 1 mean (see Coman et al., 2013). The estimated models were saturated, or ‘just identified’ (i.e., df = 0), and hence model fit indices are not meaningful. Table 2 presents the unstandardized parameter estimates from each model.

**TABLE 1:** Means, standard deviations and correlations for all variables at Time 1 (T1) and Time 2 (T2)

			Perceived COVID–19 threat		Outgroup distancing		Outgroup contact comfort		Modern racism		Ingroup attraction		Political conservatism	
		*M* (*SD*)	T1	T2	T1	T2	T1	T2	T1	T2	T1	T2	T1	T2
Perceived COVID−19 threat	T1	57.34 (26.90)	–											
	T2	50.79 (27.59)	.78**	–										
Outgroup distancing	T1	49.96 (19.05)	.26***	.27***	–									
	T2	45.96 (18.49)	.19**	.23***	.45***	–								
Outgroup contact comfort	T1	4.48 (1.58)	−.27***	−.32***	−.43***	−.38***	–							
	T2	5.48 (1.78)	−24***	−32***	−.32***	−.44***	.52***	–						
Modern prejudice	T1	2.09 (0.93)	−.12***	−.10**	.13***	.16***	−.23***	−.21***	–					
	T2	2.13 (0.96)	−.15***	−.12**	.10**	.13***	−22***	−.23***	.87***	–				
Ingroup attraction	T1	83.81 (13.98)	.01	−.05	−.07*	−.05	.09*	.12**	−.04	.01	–			
	T2	83.40 (14.28)	.02	−.04	−.04	−.08*	.06	.15***	.03	.03	.55***	–		
Political conservatism	T1	3.22 (1.36)	−.11**	−.11**	.04	.05	−.11**	−.07	.61***	.64***	.05	.08*	–	
	T2	3.30 (1.37)	−.09*	.10**	.02	.04	−.09*	−.07*	.60***	.63***	.07	.07	.90***	–

Notes

*p < .05, **p < .01, ***p < .001.

**TABLE 2:** Parameter estimates from the two‐wave BLCS models

	Model 1: COVID−19 threat and outgroup distancing			Model 2: COVID−19 threat and outgroup contact comfort			Model 3: COVID−19 threat and modern racism			Model 4: COVID−19 threat and ingroup attraction		
	*B*	*SE*	*β*	*B*	*SE*	*β*	*B*	*SE*	*β*	*B*	*SE*	*β*
Means/ intercepts												
μΔ THR	−6.30***	0.63	−.35	−6.32***	0.62	−.35	−6.30***	0.63	−.35	−6.31***	0.63	−.35
μΔ ITG	−3.94***	0.60	−.20	1.00***	0.06	.59	0.01	0.02	.02	−0.36	−0.44	−.03
Variances/residual variances												
σ^2^Δ THR	291.54***	16.70	.89	286.09***	16.54	.87	294.31***	17.11	.90	292.41***	17.00	.89
σ^2^Δ ITG	269.84***	21.00	.70	2.31***	0.11	.82	0.22***	0.02	.95	142.87***	11.57	.78
Covariances												
T1THR, T1 ITG	134.91***	19.02	.26	−11.53***	1.42	−.27	−3.05**	0.96	−.12	1.52	12.88	.004
ΔTHR, ΔITG	26.96*	10.81	.**10**	−3.88***	1.00	**−.15**	**−0.07**	0.33	**−.01**	**−12.49**	8.12	−.05
Predictive paths												
T1THR ΔTHR	−0.23***	0.02	−.34	−0.24***	0.02	−.36	−0.21***	0.02	−.32	−0.21***	0.02	−.32
T1 ITG ΔITG	−0.59***	0.04	−.57	−0.46***	0.04	−.44	−0.11***	0.02	−.22	−0.44***	0.02	−.46
T1 THR ΔITG	0.05	0.03	.07	−0.01**	0.01	−.11	−0.001*	0.001	−.08	0.01	0.02	.03
T1 ITG ΔTHR	0.09*	0.04	.10	−1.86***	0.42	−.16	−0.03	0.64	−.01	−0.10*	0.05	−.07

Boldface values in the table represent the key change‐change associations of interest. THR =Perceived COVID‐19 threat. ITG = Intergroup variable (in Model 1: outgroup distancing, in Model 2: outgroup contact comfort, in Model 3: Modern racism, Model 4: Ingroup attraction). μ_Δ_ = average change over time. σ^2^_Δ_ = variance/residual variance of change. T1 = Time 1. ΔTHR = Latent change COVID‐19 threat score. ΔITG = Latent change intergroup variable score.

*p < .05, **p < .01, ***p < .001.

#### Perceived COVID‐19 threat and outgroup distancing (Model 1)

The first model examined correlated change in perceived COVID‐19 threat and preference for physical distance from outgroup members (Table 2). Inspection of the key parameters showed that mean‐level scores on both perceived COVID‐19 threat and outgroup distancing decreased over time (as indicated by significant negative intercepts for the latent change factors). We also observed significant variability in the latent change scores for each variable, reflecting individual variability in change over time. At baseline, scores on perceived COVID‐19 threat were positively associated with outgroup distancing, as expected. Critically, there was also evidence of *correlated change* whereby the degree of reductions in perceived COVID‐19 threat significantly correlated with the degree of outgroup distancing change. That is, those with greater reductions in perceived COVID‐19 threat were, on average, those exhibiting greater reductions in outgroup distancing.

Of lesser theoretical interest, the model results also showed that higher starting values on perceived COVID‐19 threat and outgroup distancing at Time 1 were associated with smaller change in the same variable over time, whilst individuals higher in outgroup distancing at T1 showed greater reductions in threat over time.

#### Perceived COVID‐19 threat and outgroup contact comfort (Model 2)

A second model examined correlated changes in perceived COVID‐19 threat and outgroup contact comfort (Model 2, Table 2). As expected, mean‐level scores on perceived COVID‐19 threat decreased over time (i.e., a significant negative intercept for the latent change factor) and mean‐level scores on outgroup contact comfort increased over time (i.e., a significant positive intercept for the latent change factor). There was also significant interindividual variability in both change scores. Importantly, perceived COVID‐19 threat and outgroup contact comfort at baseline were significantly, negatively related, along with a significant association between change in perceived COVID‐19 threat and change in outgroup contact comfort: those who showed larger reductions in perceived COVID‐19 threat also showed larger increases in outgroup contact comfort over time.

Of lesser importance, both self‐feedback pathways were also significant – people higher in starting value of perceived COVID‐19 threat and outgroup contact comfort showed lower change in the same variables over time. Results also showed that higher starting values on outgroup contact comfort predicted lower change in perceived COVID‐19 threat over time, and higher starting values in perceived COVID‐19 threat also predicted lower change in outgroup contact comfort over time.

#### Perceived COVID‐19 threat and modern racism (Model 3)

The third model examined correlated changes in perceived COVID‐19 threat and modern racism (Model 3, Table 2). Unlike the previous two models, this model showed no significant mean‐level change in modern racism scores over time (as indicated by a non‐significant intercept for the latent change factor). Surprisingly, there was a significant negative correlation between perceived COVID‐19 threat and modern racism at baseline. That is, higher prejudice at Time 1 was associated with lower COVID‐19 threat at Time 1. No significant association was found between change in perceived COVID‐19 threat and change in levels of modern racism over time.

Again, both self‐feedback pathways were significant, with individuals scoring higher in perceived COVID‐19 threat and modern racism at Time 1 showing lower change in the same variables over time. Higher threat perceptions at Time 1 were also associated with lower change in modern racism over time.

#### Perceived COVID‐19 threat and ingroup attraction (Model 4)

The final model examined correlated changes in perceived COVID‐19 threat and ingroup attraction (Model 4, Table 2). The results showed no mean‐level change in ingroup attraction over time. Levels of perceived COVID‐19 threat and ingroup attitudes were not associated at Time 1, and there was no significant association between changes in levels of perceived COVID‐19 threat and change in levels of ingroup attraction.

Of lesser interest, the self‐feedback pathways were significant, with higher initial threat perceptions and ingroup attraction predicting lower change in the same variable over time, and higher ingroup attraction at Time 1 was also associated with lower change in perceived COVID‐19 threat over time.

### Additional analyses

**FIGURE 2:**
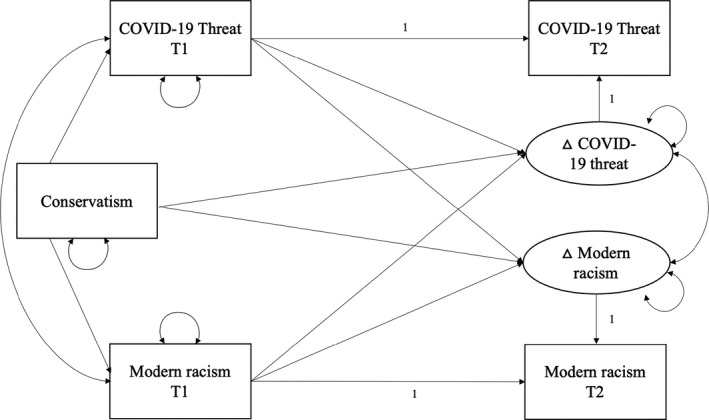
Conceptual diagram depicting an alternative BLCS model in which conservatism (Time 1) is included as a predictor of initial levels and change in perceived COVID‐19 threat and modern racism

To explore the unexpected negative baseline association between perceived COVID‐19 threat and modern racism, we tested an alternative model which included political conservatism (measured at Time 1) as a predictor of both initial levels and change in both variables (see Figure 2, for similar approach see Hadarics & Kende, 2021). Conservatism predicts higher prejudice towards a variety lower status outgroups (for review see Hodson & Dhont, 2015). But the COVID‐19 pandemic has revealed a nuanced relationship between political ideology and perceived disease threat, in part because the perception of threat has itself become politicized (e.g., concerning mask wearing or becoming vaccinated). That is, although conservatives, on average, are more threat‐sensitive than liberals (Hibbing et al., 2014; Jost et al., 2003), and more disgust‐sensitive (Terrizzi et al., 2013); conservatives are less concerned specifically about the COVID‐19 virus and report lower perceived personal vulnerability to this virus (e.g. Calvillo et al., 2020; Gollwitzer et al., 2020; Latkin et al., 2021). Accordingly, we reasoned that the negative correlation between COVID‐19 threat and modern racism observed here may be explained by the fact that politically conservative people are more likely to hold prejudiced attitudes but also more likely to deny COVID‐19 threat. Indeed, the results of the alternative model (see Model 5, Table 3) reveal that the negative baseline association between COVID‐19 threat and modern racism became non‐significant when conservatism was included in the model. Conservatism was negatively associated with perceived COVID‐19 threat at Time 1, and positively associated with modern racism at Time 1. Higher political conservatism was also associated with greater change in modern racism over time but was not predictive of the rate of change in perceived COVID‐19 threat. The results of this alternative model tested with the other intergroup variables are presented in the (see Table S2). The main pattern of results in these remaining models did not change when conservatism was included: Change in both measures of outgroup avoidance (outgroup distancing, outgroup contact comfort) remained correlated with change in perceived COVID‐19 threat (in addition to significant baseline correlations), whilst ingroup attraction remained uncorrelated with threat at either timepoint.^3^

**TABLE 3:** Parameter estimates for an alternative BLCS model including conservatism (T1) as a predictor of starting values and change in perceived COVID‐19 threat and modern racism

	Model 5: COVID−19 threat and modern racism +political conservatism		
	*B*	*SE*	*β*
Means/ intercepts			
μΔ THR	−6.30***	0.63	−.35
μΔ ITG	0.01	0.02	.01
Variances/ residual variances			
σ^2^Δ THR	293.12***	16.76	.90
σ^2^Δ MR	0.20***	0.01	.89
Covariances			
T1 THR, T1 MR	−1.32	0.75	−.07
ΔTHR, ΔMR	**0.06**	0.31	.01
Predictive paths			
T1 THR ΔTHR	−0.22***	0.02	−.32
T1 MR ΔMR	−0.21***	0.03	−.42
T1 THR ΔMR	−0.001*	0.001	−.07
T1 MR ΔTHR	0.90	0.85	.05
CON T1 THR	−2.24**	0.66	−.11
CON T1 MR	0.42***	0.02	.61
CON ΔTHR	−1.04	0.63	−.08
CON ΔMR	0.11***	0.02	.32

THR = Perceived COVID‐19 threat. MR = Modern racism. CON = political conservatism. μ_Δ_ = average change over time. σ^2^_Δ_ = variance/residual variance of change. T1 = Time 1. ΔTHR = Latent change COVID‐19 threat score. ΔMR = Latent change modern racism score.

*p < .05, **p < .01, ***p < .001.

The significance of bold value indicates the non‐significant.

## DISCUSSION

The present research took advantage of the UK’s mass COVID‐19 vaccination programme to explore evidence of a cross‐domain association between threat and outgroup prejudice. Multiple theoretical approaches in social psychology hold that perceptions of threat play a key role in understanding intergroup relations and predicting negative outgroup attitudes and treatment. Much of the existing literature relies, however, on cross‐sectional data and there have been calls for further longitudinal studies to explore threat‐prejudice associations (Riek et al., 2006). This study used a latent change modelling framework to allow us to explore the degree to which changes in perceived COVID‐19 threat and outgroup biases (prejudice, behavioural avoidance) co‐occurred amid a period of mass public vaccination in the United Kingdom. We are not aware of any studies that have directly investigated whether changes in disease threat coincide with changes in prejudice in this way, as they unfold naturally over time.

Previous research suggests that disease threat can be a particularly powerful stimulant of prejudice and discrimination against individuals perceived as ‘outsiders’ (Faulkner et al., 2004; Hodson & Costello, 2007; Navarrete & Fessler, 2006; Schaller & Neuberg, 2012; Schaller & Park, 2011). It follows from such theorizing that environmental factors that could reduce contagion threats in the context, such as mass public vaccination campaigns, may attenuate prejudicial responses. The results of this study showed that mean‐level perceptions of COVID‐19 threat indeed significantly declined during the vaccination programme, and that reductions in threat were met with reductions in outgroup avoidance. Converging results were obtained with two measures of behavioural avoidance tendencies. The first model indicated a significant correlation between change in threat and change in outgroup distancing such that individuals who showed larger reductions in perceived COVID‐19 threat over time also showed larger reductions in outgroup distancing over time. A second model then replicated this pattern with a measure of outgroup contact comfort. As expected, outgroup contact comfort was found to significantly improve over time, and change in outgroup contact comfort was positively associated with change in threat whereby those who showed larger reductions in perceived COVID‐19 threat showed larger increases outgroup contact comfort. The correspondence between rates of change in perceived COVID‐19 threat and the two measures of outgroup avoidance provides important evidence that these two variables are interrelated over time – as disease threat subsides, so too does the degree to which individuals behaviourally distance themselves from outgroup members.

Contrary to expectations, there was no significant mean‐level change in prejudiced attitudes over time, as assessed with the modern racism scale, and there was also no evidence of correlated change between prejudiced attitudes and perceived COVID‐19 threat. In the context of a contagion threat, it is perhaps not surprising that evidence of threat‐prejudice association is clearer on measures of outgroup avoidance (i.e., physical outgroup distancing, and outgroup contact comfort), than outgroup attitudes or evaluations (i.e., modern racism). Indeed, the modern racism scale is designed to measure subtle and indirect forms of prejudice. It captures the idea that prejudice against minorities is not a continuing problem, and that minorities are too demanding and have received more than they deserve (McConahay et al., 1981). The current research supports the idea that fluctuations in pathogen threats systematically related to an ‘avoidant’ psychology of negativity regarding outgroup members (Cottrell & Neuberg, 2005; Murray & Schaller, 2016; Neuberg et al., 2011), but do not necessarily coincide with more subtly racist policy attitudes.

But we also suspect that the COVID situation is somewhat unique given the politicization of the pandemic response regarding mask wearing, restrictions of movement, and vaccinations. Ordinarily threat‐ and disgust‐sensitive conservatives appear to be actively underplaying the threat by this specific virus, in line with their political leaders in many countries (see Calvillo et al., 2020; Gollwitzer et al., 2020). Moreover, Conway et al. (2021, Studies 1–4) consistently found that conservatives (*vs*. liberals) downplayed COVID‐19 threat due to motivated reasoning of a political nature (and not because of differential experiences with the virus per se). In our dataset we found that conservatism predicted higher modern racism at baseline, but also predicted lower perceived COVID‐19 threat. The negative baseline association between modern racism and perceived COVID‐19 threat become non‐significant when conservatism was included in the model. As a result, we urge some caution in interpreting the apparent lack of movement on the racism measure as a function of perceived COVID‐19 threat. This might reflect the nature of this highly politicized pandemic and might not generalize to future (or past) less politicized ones.

Of note, on measures of feelings towards fellow White Brits, and their own friends and families we found little evidence of change, and ingroup attitudes were not associated with perceived COVID‐19 threat either at baseline or over time. Such findings increase confidence that concerns about protecting oneself from disease are linked to reactions toward *outgroups*, rather than towards people generally (Huang et al., 2011). Indeed, some prior research suggests that disgust‐sensitive people seek affiliation and alliance with ingroup members as a potential source of aid and support (e.g. Hodson & Costello, 2007; Navarrete & Fessler, 2006). Yet we found no evidence of this effect here, but also found no pushing away from the ingroup. External threats were associated with aversion towards outgroup members, but not feelings of attraction towards ingroup members.

Previous research has shown that personal vaccination status (vaccinated *vs*. unvaccinated) at times moderates associations between disease primes and outgroup prejudice (Huang et al., 2011). Rather than measuring vaccine status as a between‐subjects variable, our study explored perceptions of contextual threat of COVID‐19 at two time points during a mass public vaccination programme, which saw the COVID‐19 vaccine delivered to half of all UK adults. We did run a series of alternative models in which personal vaccine status was included as a predictor of the latent change factors (see Table S1). One may expect that reductions in perceived COVID‐19 threat (and accompanying outgroup avoidance) would be largest amongst those who had personally received the vaccine during the study period, but we found no evidence of such effects here. Vaccinations are a complex phenomenon; however, and a between‐subject measure of vaccination status is potentially problematic for a number of reasons. Specifically, the rollout of the COVID‐19 vaccination programme in the United Kingdom prioritized those who were considered most clinically vulnerable (based on underlying health conditions, age etc.). Thus, participants who had been vaccinated may also be those more likely to be higher in dispositional perceptions of vulnerability. What's more, amongst unvaccinated participants would be those who had been offered but refused the vaccine because they had concerns over vaccine safety or because they denied the severity of COVID‐19. Although unprotected, such individuals may report lower threat perceptions. We also did not measure which specific vaccination individuals’ had had which may also introduce variability, especially given concerns surrounding the safety of the AstraZeneca vaccine, which were prominent in the media at the time of the second wave of data collection. Ultimately, we believe the real value in the current findings is the demonstration that perceptions of COVID‐19 threat fell amongst the population generally as the vaccine programme progressed, and such reductions in contextual threat were met with reductions in outgroup aversion.

### Limitations and future directions

As with all studies, there are some limitations of this project. Several of our key constructs, including COVID‐19 threat, were measured with a single item only. Future research should seek to replicate findings with more robust, multi‐item scales. Additionally, although we included a measure of ingroup attitudes (and found little evidence of change), we did not include a measure of avoidant behaviour towards ingroup members. Thus it is not possible to determine whether participants were uncomfortable with physical contact with immigrants specifically, or with others in general. Given the context in which the virus can be transmitted through close contact with infected persons, future research should seek to determine whether the reductions in behavioural avoidance that accompany reductions in perceived COVID‐19 threat are unique to outgroup members, or also impact behaviour towards members of one's own ingroup.

Our sample consisted of ethnic majority members in a single‐country context. It will be important to make comparisons between different countries, as well between different social groups within the same country given that data in Britain suggest that COVID‐19 vaccine hesitancy is higher amongst ethnic minority groups (Robertson et al., 2021). Moreover, data were only collected in two waves over a relatively brief period (albeit one that nicely captured the vaccine rollout). Future researchers are encouraged to explore developmental trends in perceived threat and outgroup avoidance over greater time intervals. In the United Kingdom, a 12‐week interval was initially recommended between doses. Future research should explore whether prejudicial responses may continue to decline as higher proportions of individuals become vaccinated and receive subsequent boosters.

Finally, we cannot be certain that reductions in COVID‐19 threat observed during the current study period were a direct result of vaccination programme. COVID‐19 poses a threat not only to physical health but also to economic well‐being, with many countries experiencing significant economic downturns and subsequent recoveries amid the pandemic. It is possible that other environmental factors, including economic developments, also contributed to changes in outgroup negativity observed here. Indeed, it is also possible that the intensity of people's fear of coronavirus naturally declined over time irrespective of environmental factors. Repeated exposure to fear‐inducing messages can result in desensitization to those stimuli (Hastings et al., 2004; see also Linville & Fischer, 1991). As such individuals may have become desensitized to COVID‐19 information and experienced diminished anxiety over time despite continued transmission (Stevens et al., 2021).

## CONCLUSIONS

This study used novel and robust analytic methods to provide evidence of relationships between threat and intergroup conflict. Using a latent change modelling framework, we demonstrated that perceptions of COVID‐19 threat significantly declined during a period of mass COVID‐19 vaccination in the United Kingdom. Reductions in threat were accompanied by significant reductions in outgroup distancing and significant increases in outgroup contact comfort. That is, these changes in threat and outgroup avoidance co‐occurred. By focusing on immigrants as the target outgroup, we provide evidence of an association between threat and aversion towards an outgroup that was not necessarily identified as the specific source of the threat but rather generalize toward ‘others’. These findings offer an important and timely demonstration that public health interventions that address contagion‐related threats, including vaccination programmes, may also confer other social benefits by muting psychological threat responses.

## AUTHOR CONTRIBUTIONS

**Gordon Hodson** (Conceptualization; Methodology; Writing – review & editing) **Rose Meleady**, Ph.D (Conceptualization; Formal analysis; Funding acquisition; Methodology; Project administration; Writing – original draft).

## Data Availability

The data that support the findings of this study are available from the corresponding author upon reasonable request.

## Footnotes

^1^
Some of our analyses differ from the pre‐registration. We originally planned to use individual difference measures of chronic disease threat (e.g., perceived vulnerability to disease, disgust sensitivity) in our latent change score models. In hindsight, these more time‐invariant variables were not well suited to a such a framework where the aim is to capture *change* over time. We therefore focused instead on the measure of perceived contextual disease threat. We also originally planned to include a measure of personal vaccination status in our models. Whilst we do report the results of models including vaccine status as a predictor of the latent change factors in the Supporting Information (see Table S1), they are not the main focus of our analyses due to a number of potential confounds of personal vaccine eligibility/acceptance, which may invalidate the models we originally proposed (see Discussion). Our models still test the same basic premise – that the disease protection offered by vaccinations should be associated with a reduction in outgroup negativity – but we moved away from focusing on personal immunity to exploring changes in how people feel more generally as a programme of mass public vaccination attenuated contagion threat in the environment.

^2^
It is possible to model either observed (manifest) or latent measures (multiple indicator latent change score model) of time‐dependent outcomes within the LCS model framework (McArdle, 2009). We used observed scores in estimating the LCS models, not latent variables. Measurement error could be better handled through latent modelling, but perceived COVID‐19 threat (as well as outgroup social distancing) was measured with a single item. Therefore, we report the observed score models only.

^3^
We also ran a second set of models to explore whether an individual's vaccination status influenced the rate of change in perceived COVID‐19 threat and the intergroup variables. All four BLCS models were re‐run with vaccination status (measured at Time 2) included as a predictor of the latent change scores, with vaccine status coded as a dichotomous variable (0 = unvaccinated, 1= vaccinated). Vaccine status did not significantly predict change in perceived COVID‐19 threat or any of the intergroup variables across any of the models. The full results of the alternative models including vaccination status are reported in the Supporting Information (see Table S1).
