# Supplementary material for: Reductions in perceived COVID‐19 threat amid UK’s mass public vaccination programme coincide with reductions in outgroup avoidance (but not prejudice)
Source: Br J Soc Psychol. 2022 Mar 31:10.1111/bjso.12537. Online ahead of print. doi: 10.1111/bjso.12537 (PMC9111608; doi:10.1111/bjso.12537)
Supplement: Supplementary file 1 [file BJSO-9999-0-s001.docx]

**
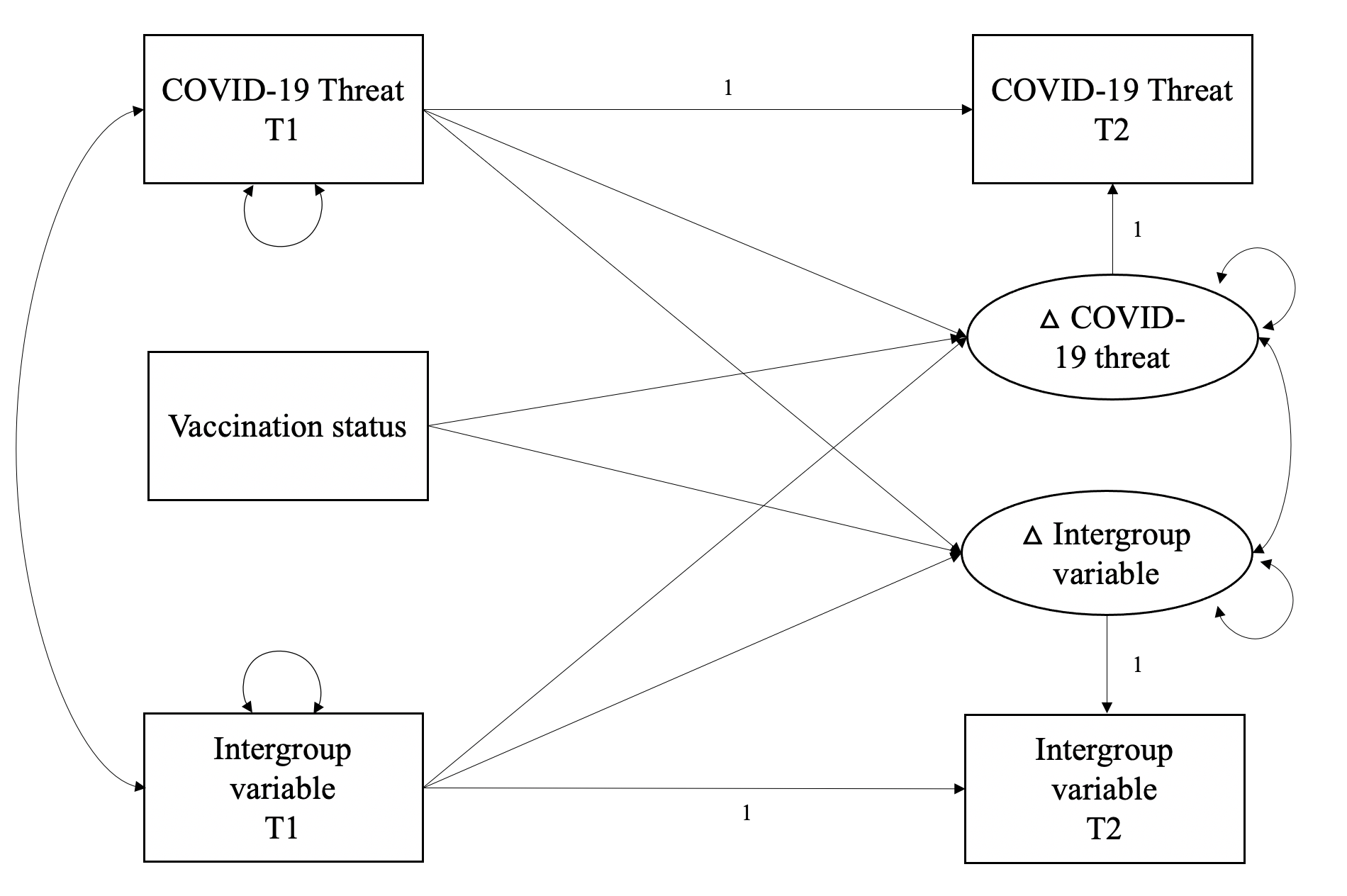
**

**Figure S1.** Conceptual diagram depicting an alternative bivariate latent change score model in which vaccination status (Time 2, 0 = unvaccinated, 1 = vaccinated) is included as a predictor of latent change factors.

**Table S1**

|  | **Model 1: COVID-19 threat and outgroup distancing** | | | **Model 2: COVID-19 threat and outgroup contact comfort** | | | | **Model 3: COVID-19 threat and modern racism** | | |  |  | **Model 4: COVID-19 threat and ingroup attraction** | | | |
| --- | --- | --- | --- | --- | --- | --- | --- | --- | --- | --- | --- | --- | --- | --- | --- | --- |
|  | ***B*** | ***SE*** | **β** | ***B*** | ***SE*** | **β** | ***B*** | | ***SE*** | **β** | ***B*** | | | ***SE*** | **β** |  |
| **Means/ intercepts** |  |  |  |  |  |  |  | |  |  |  | | |  |  |  |
| μΔ THR | -6.21*** | 0.83 | -.34 | -6.19*** | 0.87 | -.34 | -6.27*** | | 0.88 | -.01 | -6.29*** | | | 0.61 | -.34 |  |
| μΔ ITG | -3.63*** | 0.81 | -.15 | 1.00*** | 0.08 | .60 | -0.01 | | 0.02 | <.001 | -0.62 | | | -0.46 | -.05 |  |
| **Variances/ residual variances** |  |  |  |  |  |  |  | |  |  |  | | |  |  |  |
| σ^2^Δ THR | 291.53*** | 16.70 | .89 | 286.09*** | 16.52 | .87 | 294.31*** | | 17.10 | .90 | 292.41*** | | | 17.00 | .89 |  |
| σ^2^Δ ITG | 269.70*** | 21.03 | .69 | 2.31*** | 0.11 | .82 | 0.22*** | | 0.02 | .95 | 142.79*** | | | 11.56 | .79 |  |
| **Covariances** |  |  |  |  |  |  |  | |  |  |  | | |  |  |  |
| T1 THR, T1 ITG | 134.91*** | 19.02 | .26 | -11.53*** | 1.42 | -.27 | -3.05** | | 0.96 | -.12 | 1.52 | | | 12.88 | .004 |  |
| ΔTHR, ΔITG | **26.96*** | 10.81 | **.10** | **-3.88***** | 1.00 | **-.15** | **-0.07** | | 0.33 | **-.01** | **-12.49** | | | 8.12 | -.06 |  |
| **Predictive paths** |  |  |  |  |  |  |  | |  |  |  | | |  |  |  |
| T1 THR 🡪 ΔTHR | -0.23*** | 0.02 | -.34 | -0.24*** | 0.02 | -.36 | -0.21*** | | 0.02 | -.32 | -0.21*** | | | 0.02 | -.32 |  |
| T1 ITG 🡪 ΔITG | -0.59*** | 0.04 | -.57 | -0.46*** | 0.04 | -.43 | -0.11*** | | 0.02 | -.22 | -0.44*** | | | 0.02 | -.46 |  |
| T1 THR 🡪 ΔITG | 0.05 | 0.03 | .07 | -0.01** | 0.01 | -.11 | -0.001* | | 0.001 | -.08 | 0.01 | | | 0.02 | .02 |  |
| T1 ITG 🡪 ΔTHR | 0.09* | 0.04 | .09 | -1.86*** | 0.42 | -.16 | -0.03 | | 0.64 | -.001 | -0.10* | | | 0.05 | -.08 |  |
| VAX 🡪 ΔTHR | -0.18 | 1.26 | -.01 | -0.28 | 1.25 | -.01 | -0.07 | | 1.28 | -.002 | -0.05 | | | 1.27 | -.001 |  |
| VAX 🡪 ΔITG | -2.02 | 1.22 | -.05 | -0.02 | 0.11 | -.01 | 0.02 | | 0.69 | .03 | 0.59 | | | 0.89 | .02 |  |

*Parameter estimates from the two-wave BLCS models including personal vaccination status as a predictor of latent change scores*.

*Notes: THR = Perceived COVID-19 threat. ITG = Intergroup outcome (in Model 1: outgroup distancing, in Model 2: outgroup contact comfort, in Model 3: Modern racism, Model 4: Ingroup attraction). μ_Δ_, average change over time. σ^2^_Δ_, variance/residual variance of change. T1 = Time 1. ΔTHR, Latent change COVID-19 threat score. ΔITG, Latent change intergroup outcome score. VAX = vaccination status, 0 = unvaccinated, 1 = vaccinated*

**Table S2**

Parameter estimates for an alternative BLCS model including conservatism (T1) as a predictor of starting values and change in perceived COVID-19 threat and the intergroup variables.

|  | **COVID-19 threat and outgroup distancing + political conservatism** | | | | **COVID-19 threat and outgroup contact comfort + political conservatism** | | | |  | **COVID-19 threat and ingroup attraction + political conservatism** | | |  |
| --- | --- | --- | --- | --- | --- | --- | --- | --- | --- | --- | --- | --- | --- |
|  | ***B*** | ***SE*** | **β** | ***B*** | | ***SE*** | **β** | ***B*** | | | ***SE*** | **β** | |
| **Means/ intercepts** |  |  |  |  | |  |  |  | | |  |  | |
| μΔ THR | -6.28*** | 0.63 | -.35 | -6.28*** | | 0.62 | -.35 | -6.29*** | | | 0.63 | -.35 | |
| μΔ ITG | -3.96*** | 0.60 | -.20 | 0.99*** | | 0.06 | .59 | -0.38 | | | 0.46 | -.03 | |
| **Variances/ residual variances** |  |  |  |  | |  |  |  | | |  |  | |
| σ^2^Δ THR | 290.54*** | 16.44 | .89 | 284.29*** | | 16.20 | .87 | 291.77*** | | | 16.81 | .89 | |
| σ^2^Δ ITG | 269.39*** | 21.03 | .69 | 2.31*** | | 0.11 | .82 | 142.25*** | | | 11.51 | .78 | |
| **Covariances** |  |  |  |  | |  |  |  | | |  |  | |
| T1 THR, T1 ITG | 136.99*** | 18.82 | .27 | -12.05*** | | 1.41 | -.29 | 3.55 | | | 12.65 | .01 | |
| ΔTHR, ΔITG | **27.63*** | 10.75 | **.10** | **-3.94***** | | 0.99 | **-.15** | **-11.85** | | | 8.07 | -.06 | |
| **Predictive paths** |  |  |  |  | |  |  |  | | |  |  | |
| T1 THR 🡪 ΔTHR | -0.24*** | 0.02 | -.35 | -0.25*** | | 0.02 | -.37 | -0.22*** | | | 0.02 | -.32 | |
| T1 ITG 🡪 ΔITG | -0.59*** | 0.04 | -.57 | -0.46*** | | 0.04 | -.44 | -0.45*** | | | 0.04 | -.46 | |
| T1 THR 🡪 ΔITG | 0.05 | 0.03 | .07 | -0.01** | | 0.01 | -.11 | 0.02 | | | 0.02 | .03 | |
| T1 ITG 🡪 ΔTHR | 0.09* | 0.04 | .10 | -2.00*** | | 0.43 | -.18 | -0.10* | | | 0.05 | -.07 | |
| CON 🡪 T1 THR | -2.25** | 0.66 | -.11 | -2.25** | | 0.66 | -.11 | -2.25** | | | 0.66 | -.11 | |
| CON 🡪 T1 ITG | 0.50 | 0.49 | .03 | -0.13** | | 0.04 | -.11 | 0.49 | | | 0.34 | .05 | |
| CON 🡪 ΔTHR | -0.75 | 0.47 | -.06 | -1.01* | | 0.47 | -.08 | -0.60 | | | 0.48 | -.05 | |
| CON 🡪 ΔITG | 0.50 | 0.48 | .03 | -0.03 | | 0.04 | -.02 | 0.59 | | | 0.34 | .06 | |

*Notes: THR = Perceived COVID-19 threat. ITG = intergroup variable (outgroup distancing, outgroup contact comfort, or ingroup attraction). μ_Δ_ = average change over time. σ^2^_Δ_ = variance/residual variance of change. T1 = Time 1. ΔTHR = Latent change COVID-19 threat score. ΔITG = Latent change intergroup variable score. *p<.05, **p<.01, ***p<.001*
